# Effects of Insole with Toe-Grip Bar on Barefoot Balance and Walking Function in Patients with Parkinson’s Disease: A Randomized Controlled Trial

**DOI:** 10.3390/geriatrics7060128

**Published:** 2022-11-16

**Authors:** Hideki Nakano, Shin Murata, Hideyuki Nakae, Masayuki Soma, Haruhisa Isida, Yuumi Maruyama, Hitoshi Nagara, Yuko Nagara

**Affiliations:** 1 Department of Physical Therapy, Faculty of Health Sciences, Kyoto Tachibana University, Kyoto 607-8175, Japan; 2 Department of Rehabilitation, Faculty of Health Sciences, Tohoku Fukushi University, Sendai 981-8522, Japan; 3 Nagara Clinic, Fukuoka 812-0007, Japan

**Keywords:** Parkinson’s disease, toe dysfunction, insoles, balance, walking

## Abstract

The maintenance and improvement of balance and walking function in patients with Parkinson’s disease (PD) is essential. Toe dysfunction in patients with PD is related to balance and walking. Recently, insoles have been developed to improve toe function, but their effects on the physical functions of patients with PD remain unclear. In this randomized controlled study, we investigated the effects of insoles with a toe-grip bar on balance and walking function in such patients. Twenty-nine patients with PD in Hoehn and Yahr stages II–IV were randomly assigned to an intervention or control group. Patients in the intervention and control groups wore shoes having insoles with and without a toe-grip bar for 4 weeks, respectively. The center of gravity sway of standing posture (total trajectory length, envelope area, and maximum anterior–posterior center of pressure [AP-COP] distance) and walking parameters at normal and fast speeds were measured pre- and post-intervention in the rehabilitation room. All measurements were performed with the participants being barefoot. The maximum AP-COP distance and step length of the fast-walking condition were significantly improved in the intervention compared to the control group (*p* < 0.05). Thus, insoles with a toe-grip bar may improve balance and walking function in patients with PD.

## 1. Introduction

According to the Global Burden of Disease Study, neurological diseases are the leading cause of disability-adjusted life years and the second leading cause of death [1]. Parkinson’s disease (PD), one of the most common neurological diseases, affects 1.5–22 persons per 100,000 in all age groups and 529 persons per 100,000 older adults [2,3]. Furthermore, a recent meta-analysis showed that the number of patients with PD is expected to double from 6.9 million in 2015 to 14.2 million in 2040 [4]. The main motor dysfunctions of PD are balance and gait disorders, which cause falls [5]. A systematic review revealed that 60.5% of patients with PD reported at least one fall between 6 months and 1 year, while 39% reported repeated falls every year [6]. Moreover, 76% of falls in patients with PD required healthcare services, while 33% resulted in fractures [7]. Therefore, improving balance and gait would help prevent falls in patients with PD.

A recent Cochrane systematic review reported that gait, balance, and cognitive impairments are most associated with falls in patients with PD, and these may be improved with physical intervention [8]. Typical approaches to improving balance and gait impairments in patients with PD include aquatic therapy with dual task exercising, strength training, balance training, and high-frequency repetitive transcranial magnetic stimulation [9]. However, these approaches require special facilities, equipment, and continuous training. Conversely, according to a PRISMA systematic review, insoles and shoes have been reported to have positive effects on gait parameters and balance in patients with PD [10]. Insoles and shoes are simple, safe, and easy-to-use, and offer immediate results [11]; in addition, they are expected to improve the balance and walking function of patients with PD regarding toe and foot function.

According to a previous study, reduced toe-grip strength has been recognized as one of the causes of falls [12]. Toe-grip strength decreases with age [13,14], and decreased toe-grip strength is associated with balance and gait problems [15,16]. Furthermore, the toe-grip strength of individuals who have experienced a fall is lower than that of those who have not [12,17], and it is a risk factor associated with falls [17,18]. Although very few studies have investigated the toe-grip strength of patients with PD, it has been reported that their toe-grip strength is lower than that of healthy older adults and decreases with aging and disease progression [19]. Considering that the toe-grip strength in patients with PD is related to balance and gait [20,21], strengthening it may improve their balance and walking function.

In recent years, insoles with a toe-grip bar have been developed to improve toe-grip strength by walking, and they have proven to be effective [22,23,24,25,26]. These insoles can improve the toe-grip strength and toe flexibility, increase lower limb muscle activity during walking, and change spatiotemporal gait parameters in healthy individuals [22,23]. They can also improve the toe-grip strength and balance in older adults [24]. They may have similar effects in patients with PD; however, the effects of insoles with a toe-grip bar on balance and walking function in patients with PD remain unclear.

Some studies have shown that progressive resistance training [27,28], ball grasping [29,30], and towel gathering [29,31] are standard training methods for improving the toe-grip strength. However, if this training is not performed regularly, it may be difficult to maintain the beneficial effects. As a solution to this problem, insoles with a toe-grip bar have been developed recently to improve the toe-grip strength while the individual is walking [22,23,24,25,26].

The aim of this randomized controlled study was to investigate the effects of insoles with a toe-grip bar on barefoot balance and walking function in patients with PD.

## 2. Materials and Methods

### 2.1. Participants

**Figure 1:**
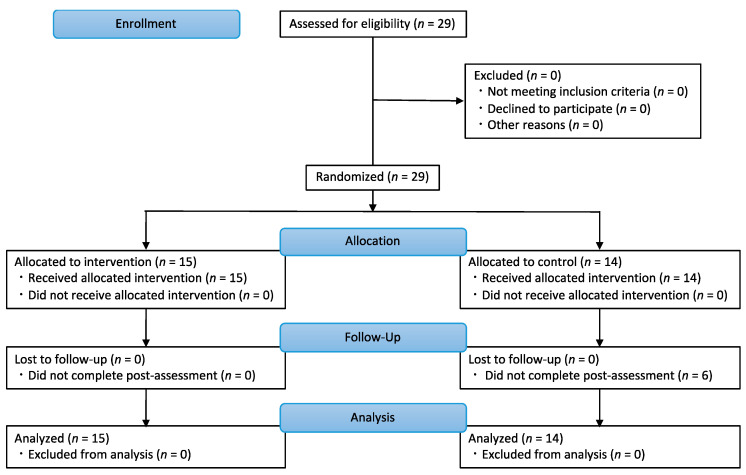
CONSORT flowchart in this study. Twenty-nine participants were randomly assigned to an intervention (*n* = 15) or control group (*n* = 14).

Twenty-nine patients with PD (mean age ± standard deviation [SD]: 71.24 ± 7.77 years; 12 men, 17 women) participated in this study. All patients were recruited from the Nagara Clinic, and they were approached as a convenience sample upon visiting the clinic. Patients with orthopedic, cardiovascular, or psychiatric diseases, which might have influenced the results, and patients who could not perform all measurements were excluded. The CONSORT flowchart is shown in Figure 1. In this study, block randomization was used to ensure a similar number of participants in each of the two groups. Participants were assigned to an intervention or control group using random numbers generated in Microsoft Excel (Microsoft, Redmond, WA, USA) based on randomization conducted by the study investigators. All the participants were blinded to the groups in which they were included. Assessors measuring balance and walking function were also blinded to the knowledge of the group the participants were assigned to.

The study was conducted according to the principles of the Declaration of Helsinki and was approved by the local institutional ethics committee (Kyoto Tachibana University, Kyoto-City, Japan). All guardians of the participants provided written informed consent, and the participants were free to withdraw from the study at any time.

### 2.2. Procedures

**Figure 2:**
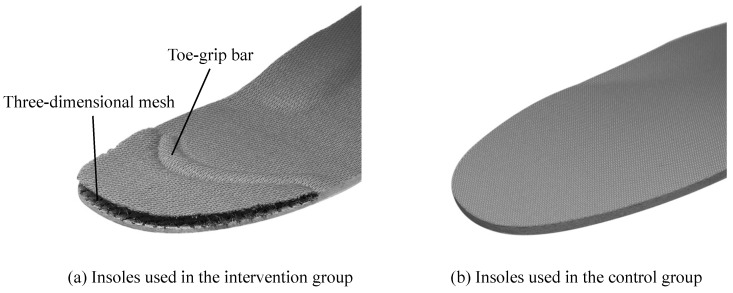
The insoles used in the intervention and control groups. (**a**) The intervention group used insoles consisting of a toe-grip bar and three-dimensional mesh. (**b**) The control group used regular insoles without a toe-grip bar.

The participants belonging to the intervention group wore shoes with insoles having a toe-grip bar [22,23,24,25,26]. The middle and rear parts of the insole were made of synthetic resin foam and the toe section was made from synthetic fibers with high repulsion properties (known as three-dimensional mesh). The toe-grip bar was placed at the center of the proximal phalanx from the first to the fifth toe (Figure 2). In this study, the appropriate insole length was selected based on the length of the participant’s foot so that the toe-grip bar was placed at the center of the proximal phalanx.

The participants in the control group wore shoes having regular insoles without the toe-grip bar or a toe section made of synthetic fibers. The structure of these insoles was the same as that of the insoles used by the intervention group. Except for the insoles, both groups wore the same standard shoes provided by the study investigators. Therefore, the foot and ankle movements were similar in both groups, and only the movements of the toes were adjusted so that they were different. Both groups of participants were instructed to use the provided shoes on a daily basis in a community-living environment; they wore the shoes 5 days per week for 4 weeks.

### 2.3. Measures

To examine the effects of the intervention, the center of gravity sway (total trajectory length [TL], envelope area [EA], and maximum anterior–posterior center of pressure [AP-COP] distance) and walking parameters (i.e., walking speed, step length, stance time, and swing time) were measured before and immediately after the 4-week intervention. All these measurements were performed with the participants being barefoot.

The center of gravity sway was measured using a stabilometer (GP-7; Anima Co., Ltd., Tokyo, Japan). The participants were instructed to stand in a two-leg stance under the following standardized conditions: barefoot, eyes open, looking at a target placed on a wall 2 m away at eye level, arms at the sides, and both feet closed [22,24,26]. The data sampling rate was 20 Hz. TL and EA were measured as indices of static balance, while AP-COP was measured as an index of dynamic balance. TL and EA were measured for 30 s after the participants had stood for 5 s to exclude the influence of the initial sway. Based on previous studies, the maximum AP-COP distance was measured by voluntarily shifting the center of gravity as far as possible in the anterior–posterior direction, according to previous studies [32,33,34]. After standing for 5 s, the participants moved their center of gravity forward as far as possible at a self-selected speed without losing their balance and held that posture for 3 s. Subsequently, they returned to the upright posture and moved their center of gravity backward in the same manner. The measurement was performed twice; the first value was excluded as a practice, whereas the second value was used for analysis.

The walking parameters (walking speed (cm/s), step length (cm), stance time (s), and swing time (s)) were assessed using a WalkWay device (WalkWay MW-1000; Anima Co., Ltd., Tokyo, Japan) [23,26], after which the temporal and spatial gait parameters were calculated based on the foot pressure distribution. It consists of a 2400 × 800 × 5 mm (length × width × thickness) mat, a sensor with a spatial resolution of 10 × 10 mm, and 14,400 measurement points. The total walking distance walked by the participants was 6.4 m, which was divided into the following sections: 2 m acceleration, 2.4 m measurement, and 2 m deceleration. The data were measured at a sampling rate of 100 Hz. The participants were instructed to walk barefoot at normal and fast speeds. The measurement was performed twice; the first value was excluded as practice, and the second value was used for the analysis.

### 2.4. Statistical Analyses

In this study, the sample size was calculated using G * Power (Universitat Dusseldorf, Dusseldorf, Germany) [35]. G * power was set as follows: test family, F tests; statistical test, analysis of variance (ANOVA); effect size, 0.40; α error probability, 0.05; power (1-β error probability), 0.80. The total sample size was calculated as 16. The baseline characteristics of the participants in the intervention and control groups were compared. The Shapiro-Wilk test was used to test the normality of distributions. Differences between the groups were analyzed using Student’s *t*-test for variables that were normally distributed and the Mann–Whitney U test for those that were not. The chi-square test was used to compare the numbers of men and women and the Hoehn and Yahr stages in each group. Measurement items were analyzed using two-way repeated ANOVA with group (intervention and control groups) and time (before and after) as factors. The Bonferroni post hoc test was used to determine which group or time periods showed significant differences. Statistical analyses were performed using IBM SPSS Statistics for Windows, version 28.0 (IBM Corp., Armonk, NY, USA). The significance level was set at <5% as the threshold for determining statistical significance. The Bonferroni correction was used for multiple comparisons such that the level of significance was 2.5%. In this study, the effect size (η^2^) was also calculated. The magnitude of the effect size was defined according to a previous study as follows: small (η^2^ = 0.01), medium (η^2^ = 0.06), and large (η^2^ = 0.14) effects [36].

## 3. Results

Before the intervention, there were no significant differences between the groups in terms of the number of men and women, number of participants belonging to each Hoehn and Yahr stage group, age, TL, EA, maximum AP-COP distance, walking speed, step length, stance time, and swing time under normal and fast-walking conditions (all *p* > 0.05, Table 1). The results of the two-way repeated measures ANOVA are shown in Table 2. There was a significant interaction in maximum AP-COP distance (F = 8.90, *p* < 0.05). Moreover, a significant interaction was observed in the step length of the fast-walking condition (F = 5.94, *p* < 0.05). There were no individuals with adverse events, such as toe pain, during or after the intervention. No individual showed freezing of gait during the pre- and post-intervention assessments in this study.

## 4. Discussion

We investigated the effects of insoles with a toe-grip bar on the balance and walking function of patients with PD. The maximum AP-COP distance and the step length when walking at the fast possible pace were significantly improved in the intervention than in the control group. These results suggest that insoles with a toe-grip bar may help improve balance and walking function in patients with PD.

Using insoles with a toe-grip bar for 4 weeks increases toe-grip strength in healthy individuals and older adults [22,24]. Therefore, these insoles might have strengthened toe-grip in patients with PD in this study. The toes play an essential role in the control of forward movement during walking [37,38], and the stride length while walking at a fast pace decreases in older adults with reduced toe-grip strength [39]. Furthermore, there is a significant relationship between toe-grip strength and step length while walking at a fast pace in patients with PD [40]. Thus, improved toe-grip strength when using insoles with a toe-grip bar might have increased the step length while walking at a fast pace in patients with PD.

The center of gravity sway results in this study showed that the maximum AP-COP distance, which reflects dynamic balance, was significantly better in the intervention than in the control group; however, there were no significant differences between the groups in TL and EA, which reflect static balance. The toes play an essential role in the control of postural balance [41,42,43]. Moreover, postural sway increases and the maximum balance range decreases in older adults with reduced toe-grip strength [15]. A recent systematic review demonstrated that strengthening toe-grip improves postural balance [44]. Additionally, in our preliminary study on patients with PD, we found that the use of insoles with a toe grip bar shifted the center of foot pressure position forward and increased the maximum AP-COP distance more than when standing barefoot [45]. These results suggest that improving toe-grip strength by using insoles with a toe-grip bar promoted dynamic balance in patients with PD.

Although the detailed mechanism of the effect of the insoles with a toe-grip bar is unknown, one of the reasons for the improvement effect is thought to be the increase in toe movement by the toe-grip bar. The insoles used in this study were composed of a toe-grip bar made from synthetic fiber with high repulsive properties. In the terminal stance of walking, the toes perceive the toe-grip bar and the reflex toe-grip movement increases. Previous studies have reported that exercise of toe-grip movement is effective for increasing toe-grip strength in older people [22,23,24,25,26]. Our results suggest that toe-grip movement occurs subconsciously when the individual walks, resulting in improved balance and walking function related to toe-grip strength [19,20,39,44].

PD is a progressive disease; therefore, regular exercise is essential to maintain and improve balance and gait function. However, regular exercise is affected by individual, environmental, and activity characteristics [46]. However, this study showed that just walking using the insole with a toe-grip bar improved balance and gait function of patients with PD. Thus, such insoles can be used continuously and efficiently in daily life without the need to perform any particular exercise and are expected to be widely used.

Recent systematic reviews have reported that typical approaches to improve balance and walking function in patients with PD include aquatic therapy with dual task exercising, strength training, balance training, and high-frequency repetitive transcranial magnetic stimulation [9]. The PRISMA systematic review also found that insoles and shoes have positive effects on gait parameters and balance in patients with PD [10]. Interventions using insoles and shoes are a simple and safe approach that can be implemented simply by walking. Therefore, it is expected to be one of the most useful tools for patients with PD.

This study has some limitations. First, the time for which the participants used the provided shoes and the number of steps they took during the intervention period were not measured. Therefore, the relationship between the time spent using the provided shoes or the number of steps and change in performance is not clear. In future studies, these parameters must be measured to elucidate these associations. Second, the toe-grip strength was not measured; therefore, it remains unknown whether it increased after the intervention. The relationship between increased toe-grip strength and improved balance and walking function is also unclear. In the future, it is necessary to verify whether insoles with a toe-grip bar directly improve the toe-grip strength of patients with PD. In addition, pressure-sensored insoles could be used to monitor the impact of the toe-grip bar and to observe the improvement in pressure after 4 weeks. Third, we could not examine how long the effect of the intervention lasted. Therefore, it is unclear whether the insoles with a toe-grip bar must be used continuously to maintain the benefits. The persistence of the effects of the interventions in patients with PD should also be examined in future studies. Fourth, we could not control whether the participants performed other physical training in their daily lives. Future studies should examine the effects of training in daily life while controlling for the presence or absence of training. Fifth, the insoles were not created and were fully adjusted to the characteristics of the participant’s feet. Therefore, it is necessary to verify the effectiveness of the insole by using an insole that is fully adjusted to these characteristics. Sixth, the measurement distance for walking was 2.4 m. Future research should measure gait parameters over longer distances. Seventh, the effect of insoles with a toe-grip bar on freezing of gait was not examined. Future studies should examine the effects on freezing of gait from multiple perspectives.

## 5. Conclusions

We investigated the effects of insoles with a toe-grip bar on balance and walking function in patients with PD. The maximum AP-COP distance and the step length under fast-walking conditions were more significantly improved in the intervention group than those in the control group. Thus, insoles with a toe-grip bar may improve balance and walking function in patients with PD. This insole could be a simple and safe tool to improve the balance and walking function of patients with PD.

## Figures and Tables

**Table 1:** Characteristics of the intervention and control groups.

Parameters		Intervention Group (*n* = 15)	Control Group (*n* = 14)	*p*-Value
Men/Women (*n*)		5 /10	7 / 7	0.36
Hoehn and Yahr stage (n)	II	2	1	0.86
	III	11	11	
	IV	2	2	
Age (years)		71.13 ± 7.91	71.36 ± 7.91	0.94
Total trajectory length (cm)		47.58 ± 27.43	55.96 ± 25.31	0.40
Envelop area (cm^2^)		2.85 ± 1.80	3.60 ± 3.80	0.50
Maximum AP-COP distance (cm)		7.88 ± 2.46	9.24 ± 2.55	0.16
Normal walking condition	Walking speed (cm/s)	83.14 ± 24.22	82.45 ± 22.33	0.94
Fastest walking condition	Step length (cm)	42.70 ± 10.62	45.79 ± 9.95	0.43
	Stance time (s)	0.74 ± 0.12	0.77 ± 0.07	0.56
	Swing time (s)	0.33 ± 0.04	0.36 ± 0.03	0.42
	Walking speed (cm/s)	125.31 ± 34.63	138.26 ± 29.34	0.29
	Step length (cm)	53.21 ± 12.98	59.55 ± 9.92	0.15
	Stance time (s)	0.57 ± 0.08	0.59 ± 0.06	0.60
	Swing time (s)	0.27 ± 0.07	0.29 ± 0.04	0.43

Values are expressed as mean ± SD; AP-COP, Anterior-posterior center of pressure.

**Table 2:** Comparison of parameters before and after training in the intervention and control groups.

Parameters		Group	Before		After		95% CI	Group × Time		ES
			Mean	SD	Mean	SD		*F*-Value	*p*-Value	η^2^
Total trajectory length (cm)		Intervention	47.58	27.43	43.11	16.46	−7.44–16.39	0.00	0.96	0.00
		Control	55.96	25.31	51.11	21.91	−7.48–17.19			
Envelop area (cm^2^)		Intervention	2.85	1.80	2.59	1.87	−1.11–1.63	0.70	0.41	0.03
		Control	3.60	3.80	2.53	1.05	−0.35–2.48			
Maximum AP-COP distance (cm)		Intervention	7.88	2.46	9.75	2.48	0.85–2.89	8.90	0.01 *	0.25
		Control	9.24	2.55	8.98	2.45	−1.31–0.79			
Normal walking condition	Walking speed (cm/s)	Intervention	83.14	24.22	98.27	23.60	6.12–24.13	1.50	0.23	0.05
		Control	82.45	22.33	89.83	26.28	−1.94–16.70			
	Step length (cm)	Intervention	42.70	10.62	49.14	10.99	2.52–10.36	1.48	0.23	0.05
		Control	45.79	9.95	48.89	12.73	−0.96–7.15			
	Stance time (s)	Intervention	0.74	0.12	0.68	0.07	0.02–0.10	2.48	0.13	0.08
		Control	0.77	0.07	0.75	0.08	−0.02–0.06			
	Swing time (s)	Intervention	0.33	0.04	0.33	0.03	−0.02–0.02	0.36	0.55	0.01
		Control	0.36	0.03	0.35	0.04	−0.02–0.03			
Fastest walking condition	Walking speed (cm/s)	Intervention	125.31	34.63	138.98	37.92	0.31–27.04	3.21	0.08	0.11
		Control	138.26	29.34	135.14	41.77	−16.96–10.71			
	Step length (cm)	Intervention	53.21	12.98	58.76	12.82	1.43–9.67	5.94	0.02 *	0.18
		Control	59.55	9.92	58.05	15.15	−5.77–2.77			
	Stance time (s)	Intervention	0.57	0.08	0.58	0.08	−0.03–0.03	0.02	0.90	0.00
		Control	0.59	0.06	0.59	0.07	−0.03–0.03			
	Swing time (s)	Intervention	0.27	0.07	0.29	0.05	−0.01–0.05	0.57	0.46	0.02
		Control	0.29	0.04	0.29	0.04	−0.03–0.04			

SD, Standard deviation; AP-COP, Anterior-posterior center of pressure; CI, Confidence interval; ES, Effect size; * *p* < 0.05, Significant difference before and after the intervention.

## Data Availability

The data used to support the findings of this study are available from the corresponding author upon request. The data are not publicly available because they contain information that can compromise the privacy of research participants.
